# Reviewing the complex link between epilepsy and psychosis. A case report

**DOI:** 10.1192/j.eurpsy.2021.2152

**Published:** 2021-08-13

**Authors:** P. Albarracin, M. Jiménez Cabañas, E. Herrero, R. Galeron, M. Huete Naval, A. Garcia Recio

**Affiliations:** 1 Psychiatry And Mental Health, Hospital Clínico San Carlos, Madrid, Spain; 2 Psychiatry And Mental Health, Hospital Clinico San Carlos, Madrid, Spain

**Keywords:** psychosis, epilepsia, schizophrénia, bipolar

## Abstract

**Introduction:**

We present the case of a 56 year old woman with a diagnose of Complex Partial Seizures since her teenage years, with a history of multiple hospitalizations in the psychiatric ward of our hospital and a challenging clinical evolution.

**Objectives:**

To review the different kinds of psychotic disorders that may arise in relation to epilepsia.

**Methods:**

Literature review of scientific papers and classic textbooks on the issue, including references in both Spanish and English languages.

**Results:**

From 2008 to 2011 our patient was hospitalized with episodes of different clinical features leading to different diagnoses (in 2008 the episode was compatible with a maniac phase and led to a diagnosis of possible Bipolar Disorder, in 2010 dissociative-like symptoms became more prominent and led to a diagnose of Dissociative Identity Disorder and in 2011 the symptoms pointed to an interictal depression), and a subsequent symptomatology that made clinicians consider a diagnose of unspecified schizophrenia. From 2015 to 2020 our patient suffered multiple decompensations resulting in up to six new hospitalizations, with psychotic symptoms in the shape of auditive hallucinations being consistent and affective symptoms varying widely. This evolution suggests a plausible diagnose of interictal chronic psychosis with bipolar-like affective episodes.

**Conclusions:**

An extensive review of the available scientific literature shows, as so does this case, that along the course of an epileptic disease both schizophrenia-like psychosis and affective psychosis may arise, and that those might be divided along the categories of peri ictal and inter ictal disorders.

**Disclosure:**

No significant relationships.

